# Independent and Combined Effects of All-Out Sprint and Low-Intensity Continuous Exercise on Plasma Oxidative Stress Biomarkers in Trained Judokas

**DOI:** 10.3389/fphys.2019.00842

**Published:** 2019-07-09

**Authors:** Kais El Abed, Achraf Ammar, Omar Boukhris, Khaled Trabelsi, Liwa Masmoudi, Stephen J. Bailey, Ahmad Hakim, Nicola Luigi Bragazzi

**Affiliations:** ^1^Research Unit of Pharmacology and Toxicology of Xenobiotics (UR12 ES13), Faculty of Medicine, University of Sfax, Sfax, Tunisia; ^2^UR15JS01: Education, Motricité, Sport et Santé (EM2S), High Institute of Sport and Physical Education, University of Sfax, Sfax, Tunisia; ^3^Institute of Sport Science, Otto von Guericke University Magdeburg, Magdeburg, Germany; ^4^Unit of Research Molecular Bases of Human Diseases, Faculty of Medicine of Sfax, University of Sfax, Sfax, Tunisia; ^5^School of Sport, Exercise and Health Sciences, Loughborough University, Loughborough, United Kingdom; ^6^Department of Health Sciences (DISSAL), Postgraduate School of Public Health, University of Genoa, Genoa, Italy; ^7^Department of Mathematics and Statistics, Laboratory for Industrial and Applied Mathematics (LIAM), York University, Toronto, ON, Canada

**Keywords:** redox biomarkers, physical exercise, cycle ergometer, physiological responses, response to exercise

## Abstract

The purpose of this study was to assess oxidative stress biomarkers prior to and following different forms of exercise. Ten elite male judokas (age: 18.1 ± 1.7 years, athletic experience: 6 years with a training frequency of 6 Judo-sessions/week) performed three cycle ergometry sessions comprising a 30 s Wingate test (MAX), 30 min at 60% maximal-aerobic-power-output (LOW) or these two exercise protocols combined (COMBINED) in a repeated-measures design. Venous blood-samples were collected before, and 0(P0), 5(P5), 10(P10) and 20(P20) min after each exercise protocol and assessed for malondialdehyde concentration ([MDA]), glutathione peroxidase (GPX), superoxide dismutase (SOD) and glutathione reductase (GR) content, and total-antioxidant-status (TAS). Plasma [MDA] was found to be increased above baseline at P0 and P5 in the MAX, LOW and COMBINED conditions (*p* < 0.05), but was greater at P10 and P20 in the LOW condition compared to MAX and COMBINED conditions (*p* < 0.05). Blood GPX and SOD content increased above baseline at P0 in MAX and COMBINED and at P5 in LOW (*p* < 0.05), with GR content being similar between groups at P0 and P5 (*p* > 0.05). 20 min post-exercise, GPX, SOD, GR content and TAS were lower in the MAX compared to the LOW and COMBINED conditions (*p* < 0.05). In conclusion, the findings from this study reveal that redox-related biomarkers exhibited divergent response dynamics following different forms of exercise, which might have implications for understanding the mechanisms of exercise-induced skeletal muscle fatigue and adaptive remodeling.

## Introduction

Reactive oxygen species (ROS) are free radical molecules that can oxidatively modify and damage cellular constituents including lipids, proteins and DNA (Leeuwenburgh and Heinecke, 2001; Bloomer et al., 2007). In healthy adults at rest, ROS-mediated oxidative damage is mitigated by a variety of enzymatic [e.g., superoxide dismutase (SOD), catalase and glutathione peroxidase (GPX)] and non-enzymatic (e.g., glutathione, ascorbic acid, and α-tocopherol) antioxidants (Matés et al., 1999). However, in certain disease conditions (Bloomer and Goldfarb, 2004), and during exercise of a sufficient duration and intensity (Alessio et al., 1988; Kayatekin et al., 2002; Baker et al., 2004; Ammar et al., 2015a, 2016a,b), increased ROS production can outweigh antioxidant systems leading to the development of oxidative stress (Finaud et al., 2006; Powers and Jackson, 2008; Ammar et al., 2015a, 2016a, 2017b). Although increased ROS production and the ensuing development of oxidative stress have historically been considered noxious processes that impair human health and function, their prevailing effect on human health and performance remains controversial (Fisher-Wellman and Bloomer, 2009; Powers et al., 2016).

Although, the development of exercise-induced oxidative stress is well documented during different types of exercise, including low-intensity continuous (Wadley et al., 2016), high-intensity interval (Child et al., 1998; Wadley et al., 2016; Parker et al., 2018) and all-out sprint (Marzatico et al., 1997; Parker et al., 2018) exercise, it is presently unclear which type of exercise elicits the greatest oxidative stress response. Indeed, the few studies that have directly compared the effects of short-duration high-intensity exercise bouts and continuous low-intensity exercise bouts on redox balance have yielded disparate findings (Marzatico et al., 1997; Bloomer et al., 2005; Wadley et al., 2016; Parker et al., 2018). Importantly, it appears that when short-duration ≤30 s maximal intensity “all-out” exercise is compared to longer duration lower-intensity continuous exercise, some prooxidant and antioxidant markers are increased to a greater extent in the former (Parker et al., 2018). However, when high-intensity intervals are of a submaximal intensity, post-exercise oxidative stress biomarkers are similar compared to lower-intensity continuous exercise (Wadley et al., 2016). Therefore, exercise intensity appears to be an important stimulus for regulating post-exercise oxidative stress biomarkers. However, the effect of combining short-duration high-intensity and continuous low-intensity exercise bouts in a single exercise session has yet to be investigated. Resolving which type of exercise elicits the greatest oxidative stress response is important as this might reveal the exercise settings with the greatest potential to exhibit improved performance following antioxidant supplementation (Ammar et al., 2016b, 2018), and to elicit the fastest or greatest improvement in physiological and performance adaptations following a chronic exercise training intervention (Ammar et al., 2017a).

Although Parker et al. (2018) recently showed that maximal-intensity sprint interval exercise elicited a greater post-exercise hydrogen peroxide production and CAT activity compared to continuous moderate-intensity exercise, Marzatico et al. (1997) and Inal et al. (2001) demonstrated that the activities of enzymatic antioxidants increased by a similar magnitude following low- and high-intensity exercise. These discrepancies could be attributable to the inter-study differences in the exercise training status of the participants (Ammar et al., 2015a, 2016a, 2017a). Indeed, it is well documented that chronic exercise training augments the antioxidant systems by increasing the production of endogenous antioxidants (GPX, CAT, SOD, and GR; Powers and Jackson, 2008). Consistent with this, recent evidence suggests that high-intensity resistance training can suppress or prevent the increase in [MDA] after short-duration high-intensity exercise (Bloomer et al., 2005; Bloomer et al., 2007; Ammar et al., 2015a, 2016a, 2017a). Therefore, it is important to determine the effect of different forms of exercise on the development of oxidative stress in well-trained subjects as these participants might exhibit divergent responses in post-exercise oxidative stress biomarkers compared to their lesser trained counterparts. As a result, elite Judokas were recruited as participants in the current study as they are accustomed to performing different types of exercise (including continuous low-intensity exercise, short-duration maximal exercise and the combinations of these types of exercise) in their regular training program.

The purpose of the current study was to compare the effects of three different acute exercise sessions, consisting of a 30 s all-out sprint (MAX), 30 min of low-intensity continuous exercise (LOW) and these two exercise protocols combined (COMBINED), on post-exercise oxidative stress biomarkers in elite Judokas. Given that there is a lack of consensus on the most accurate oxidative stress biomarker, the current study assessed exercise-induced changes in a variety of oxidative stress biomarkers to facilitate a more robust evaluation of exercise-induced oxidative stress, in line with previous recommendations (Powers and Jackson, 2008; Ammar et al., 2017a, 2018; Cobley et al., 2017). Specifically, [MDA] was assessed as a marker of lipid peroxidation (Bloomer, 2008), the content of the antioxidant enzymes, GPX, SOD, and GR, were assessed as they are increased in response to elevated ROS production in an attempt to mitigate oxidative stress development (Matés et al., 1999), and the plasma concentration of the non-enzymatic antioxidant, α-tocopherol, was assessed as it declines in conditions of increased ROS production (Groussard et al., 2003; Ammar et al., 2015a, 2017b). Since some oxidative stress biomarkers have been reported to be altered to a greater extent following all-out sprint exercise compared to continuous submaximal exercise (Parker et al., 2018), we hypothesized that the series of oxidative stress biomarkers employed in the current study would be perturbed to a greater extent post-exercise in the MAX condition compared to the LOW condition. In addition, and on the basis that ROS production has been reported to increase in an intensity and duration-dependent way during exercise (Reid, 2016), we hypothesized that a greater degree of oxidative stress would be manifest post-exercise in the COMBINED compared to the MAX and LOW conditions.

## Materials and Methods

### Participants

Ten elite male judokas participating in a regional Judo team and competing at an international standard [mean ± SD age: 18.1 ± 1.7 years, body mass: 77.2 ± 11.7 kg, height: 1.76 ± 0.05 m, peak oxygen uptake (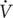
O_2peak_): 51.2 ± 8.4 mL⋅kg^−1^⋅min^−1^] volunteered to participate in this study. The participants were recruited on the basis that they are aged between 18 and 24 years old, had a BMI less than 25 kg/m^2^, were not regular creatine users and had more than 6 years judo experience with a frequency of six training sessions (1.5–2 h) per week. Typically, the training sessions consisted of a 20-min warm-up, 25 min Uchi-Komi, 10 min Nage-Komi, 20 min Randoris (Ne waza), 15 min Randoris and 15 min strength and stretching exercises. Additionally, participants were required to avoid consuming foods rich in antioxidants and polyphenols during the experimental period and the preceding 3 weeks and to avoid strenuous exercise during the experimental period. Participants provided written informed consent to participate in the study. The study was conducted according to the declaration of Helsinki with the protocol fully approved by the Sfax University Ethics Committee (ID: 8/12) before the commencement of the study.

### Experimental Design

One week before the start of the experimental period, 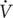
O_2peak_ and maximal aerobic power output (MAP) were determined for each participant from an incremental laboratory cycling test (González-Haro et al., 2007). After a 10-min warm-up at 100 W, the test began at an initial power output of 200 W. Subsequently, power output was increased by 30 W every 4 min until RER≥1. Thereafter, power output was increased by 10 W/min until exhaustion. During the test, 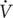
O_2_ was measured breath by breath using an indirect calorimetry system (Quark PFT, Cosmed, Rome, Italy) (González-Haro et al., 2007). MAP was calculated using the equation proposed by Kuipers et al. (1985). The 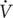
O_2peak_ was determined from the mean 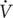
O_2_ over the last 30 s of the test.

As part of a repeated-measures, cross-over experimental design, participants performed three randomized test sessions interspersed by a recovery period of 72 h to allow sufficient recovery of oxidative stress biomarkers (Ammar et al., 2015a). Additionally, to avoid any time of day effects, all sessions were conducted in the early evening hours, as suggested by Ammar et al. (2015b, 2017b). The testing sessions required the completion of either MAX, LOW, or COMBINED exercise protocols.

Upon arrival for their first test session, each participant’s body mass (Tanita, Tokyo, Japan) and height were recorded. Before completing the experimental testing sessions, a standardized 5 min cycling warm-up was completed at 75 W. The MAX protocol comprised a single standard 30 s Wingate test on an electronically braked cycle ergometer (Excalibur Sport, Lode B.V, Medical Technology, Groningen, Netherlands) connected to a computer with diagnostic software (Ergocard^®^, Medisoft, Dinant, Belgium). Following the warm-up, participants were instructed to pedal as fast as possible during a 6 s acceleration phase to attain peak cadence. Immediately following the acceleration phase, the load was electronically applied to the flywheel and subjects pedaled “all-out” for the entirety of 30 s. The LOW protocol consisted of pedaling on the same cycle ergometer at an intensity equal to 60% of MAP for a duration of 30 min at a cadence of 60 rpm. The COMBINED protocol involved the completion of the MAX protocol followed by the LOW protocol with 3 min passive recovery between these protocols. Immediately prior to (rest) and following (P0) each testing session, as well as 5 min (P5), 10 min (P10) and 20 min (P20) following each exercise session, blood samples were collected from a forearm vein through an intravenous cannula. Additionally, ratings of perceived exertion (RPE) were measured immediately following each test session.

### Ratings of Perceived Exertion Scale

The RPE scale allows participants to give a subjective exertion rating for a physical task (Borg, 1982). The participants were familiarized to the use of the RPE scale. The scale presents a 15-point scale ranging from 6 (very very light) to 20 (very very hard). The RPE scale is a reliable indicator of physical discomfort, has sound psychometric properties, and is strongly correlated with several other physiological measures of exertion (Chtourou et al., 2012).

### Dietary Records

To assess the adequacy and consistency of nutrient intake, a daily dietary record was completed over 7 days. All participants received detailed verbal and written instructions on the process of recording their diet. Participants were asked to continue with their usual dietary habits during the period of dietary recording and to be as accurate as possible in recording the amounts and types of food and fluid consumed. A list of common household measures (e.g., tablespoons, cups), and specific information about the quantity in each measurement (grams, etc.) was given to each participant. The individuals diet was evaluated using the Bilnu 4 software (SCDA Nutrisoft, Cerelles, France) and the food composition tables published by the Tunisian National Institute of Statistics in 1978. Estimated nutrient intakes were compared to reference dietary intakes for physically active people and the daily nutriment data showed that total calorie, macronutrient, and micronutrient intakes were within the reference dietary intakes for healthy Tunisian adults with no significant differences between the three test sessions (Table 1).

**Table 1:** Dietary record of the subjects (mean ± SD).

Variables	Mean ± SD		
	Session 1	Session 2	Session 3
**Kilocalorie (day^−1^)**	3271 ± 485	3244 ± 510	3303 ± 590
**Carbohydrates (%)**	53.03 ± 5.9	51.54 ± 5.1	52.26 ± 5.3
**Protein (%)**	11.93 ± 1.2	12.67 ± 1.5	12.11 ± 1.7
**Fat (%)**	27.09 ± 4.0	28.28 ± 4.8	27.46 ± 3.9
**Cholesterol (mg⋅day^−1^)**	324.1 ± 98	310.9 ± 76	350.7 ± 81
**Vit C (mg⋅day^−1^)**	45.33 ± 11	43.34 ± 09	44.97 ± 13
**Vit E (mg⋅day^−1^)**	4.11 ± 0,9	4.04 ± 0,7	4.17 ± 1,1
**Vit A (ER)**	1300 ± 252	1270 ± 198	1320 ± 203
**Folate (μg⋅day^−1^)**	338.8 ± 63	329.6 ± 54	345.4 ± 71
**Vit B12 (μg⋅day^−1^)**	7.2 ± 2.1	6.9 ± 1.8	7.4 ± 2.4

### Blood Analysis

Blood samples were taken from an antecubital vein into a vacutainer via venipuncture. Samples were immediately centrifuged for 5 min at 4,000 rpm at 4°C to obtain plasma. To eliminate inter-assay variance, all samples were analyzed in duplicate, in the same assay run, and in the same laboratory. SOD, GPX and GR content, and TAS were measured using standard colorimetric assays (Randox Laboratories Limited, 55 Diamond Road, Crumlin, County Antrim, BT29 4QY, United Kingdom) as described below.

#### SOD

After recovery of plasma, packed red blood cells were washed 4 times with 3 ml of 9% NaCl and centrifuged for 10 min at 3,000 rpm after each wash. Cell lysis was performed by adding 2 ml of cold double-distilled water. After the red blood cells rested for 15 min at 4°C, the hemolysate was diluted 1:50 with a 0.01 M phosphate buffer, pH 7. The reaction was carried out at 37°C, and the optical density was read at 505 nm. A first reading was performed 30 s after the start of the reaction, and a second reading was performed after 3 min 30 s. The results were calculated using the formulas described by El Abed et al. (2011). A standard curve was constructed on semilogarithmic paper by increasing the percentage of inhibition of standards for subsequent calculation of SOD content (units SOD per milliliter). Intra- and inter-assay coefficient of variation for the SOD were 0.8% and 0.9%.

#### GPX

A 0.05 mL sample of heparinized whole blood was prediluted with 1 mL of diluent supplied in the kit to convert oxidized glutathione (GSSG) to its reduced form (GSH). After 5 min of incubation, the sample was diluted with 1 mL of Drabkin’s reagent to inhibit interference from other peroxidases present in the sample. The samples were assessed within 20 min of the addition of Drabkin’s reagent. The decrease in absorbance was measured at 340 nm. Three readings were recorded 1, 2, and 3 min from the start of the reaction. The GPX concentration was calculated from the formula described by El Abed et al. (2011). Intra- and inter-assay coefficient of variation for the GPX were 0.9 and 1.0%.

#### GR

After recovery of plasma, red blood cells were washed 3 times with 3 mL of NaCl 9% and centrifuged for 5 min at 2,000 rpm after each wash. Cell lysis was performed by adding 0.5 mL of cold double distilled water. After 10 min incubation at 4°C, the hemolysate was centrifuged for 5 min at 2,000 rpm to remove the stroma. Subsequently, 100 mL of hemolysate was diluted with 1.9 mL of 9% saline. The absorbance was read at 340 nm 1, 2, 3, 4, and 5 min from the beginning of the reaction. GR activity was calculated from the formula described by El Abed et al. (2011). Intra- and inter-assay coefficient of variation for the GR were 0.7 and 0.8%.

#### TAS

After incubation of the reaction mixture consisting of the sample, standard or a blank and a chromogen at 37°C, the initial absorbance was read at 600 nm. The second reading was made exactly 3 min after adding the hydrogen peroxide (H_2_O_2_) substrate. TAS was calculated as described by El Abed et al. (2011). Intra- and inter-assay coefficient of variation for the TAS were 0.6 and 0.7%.

#### α-Tocopherol

α-tocopherol was extracted with hexane from human plasma and then measured via high performance liquid chromatography (HPLC) as described by Siluk et al. (2007). For specimen preparation, 100 μL of internal standard and 100 μL of plasma were mixed for 5 s. Subsequently, 200 μL of ethanol was added and mixed for 30 s, followed by 500 μL of hexane which was mixed for 1 min. The mixture was centrifuged at 4000 rpm and 4°C for 8 min with 450 μL of the supernatant removed after centrifugation and evaporated to dryness under a stream of nitrogen at room temperature. Solids were taken by the addition of 250 μL of methanol, vortexing for 30 s followed by centrifugation (4000 rpm and 4°C for 8 min) before finally analyzing using the HPLC method described by Siluk et al. (2007). Intra- and inter-assay coefficient of variation for the α-tocopherol were 1.1 and 1.2%.

#### MDA

MDA was assessed as a marker of lipid peroxidation using a colorimetric reaction, which uses 1-methyl-2-phenylindole as a chromogen (McCusker et al., 1993). Condensation of one molecule of MDA with 2 molecules of 1-methyl-2-phenylindole under acidic conditions results in the formation of a chromophore with an absorbance maximum at 586 nm. A 7.6 mM solution of 1-methyl-2-phenylindole (MPI) was prepared immediately prior to use, in 33% methanol in acetonitrile. A 650 μL aliquot of MPI was placed in each test tube, followed by the addition of 200 μL of plasma. The tubes were mixed well, and 150 μL of 10 M HCl was added. After mixing once more, tubes were sealed, and incubated for 60 min at 45°C. After incubation, tubes were chilled on ice and centrifuged at 10,000 × *g* for 5 min to remove debris. The absorbance at 586 nm was subsequently measured and subtracted from the blank value, obtained by replacing plasma with water. A calibration plot was prepared using 4, 8, 16, and 20 μmol/L of 1,1,3,3-tetramethoxypropane in 20 mM Tris–HCl, buffer, pH 7.4 (McCusker et al., 1993). Intra- and inter-assay coefficient of variation for the MDA were 1.6 and 1.7%.

### Statistical Analysis

**FIGURE 1:**
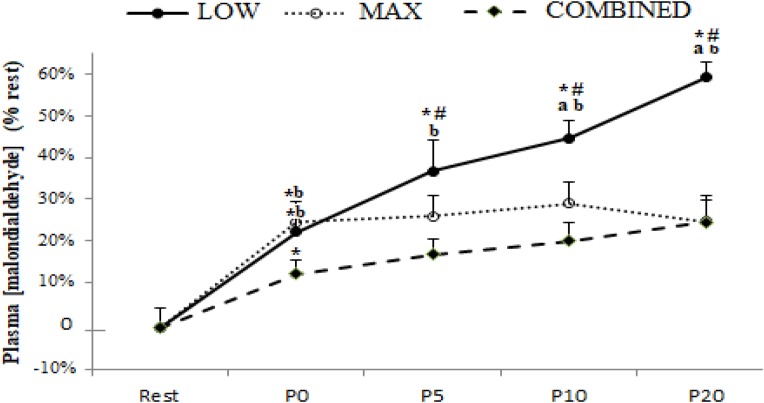
Plasma malondialdehyde concentration before (Rest), immediately after (P0), and 5 (P5), 10 (P10) and 20 (P20) minutes after maximal-intensity, low-intensity and maximal- and low-intensity exercise combined. Data are expressed as the % change from pre-exercise resting concentrations. LOW: low-intensity exercise; MAX: maximum-intensity exercise; COMBINED: combined maximum-intensity and low-intensity exercise; ^∗^: significant difference compared to pre-test values; # significant difference compered to P0, a: significant difference compared to the MAX exercise;b: significant difference compared to the COMBINED exercise.

**FIGURE 2:**
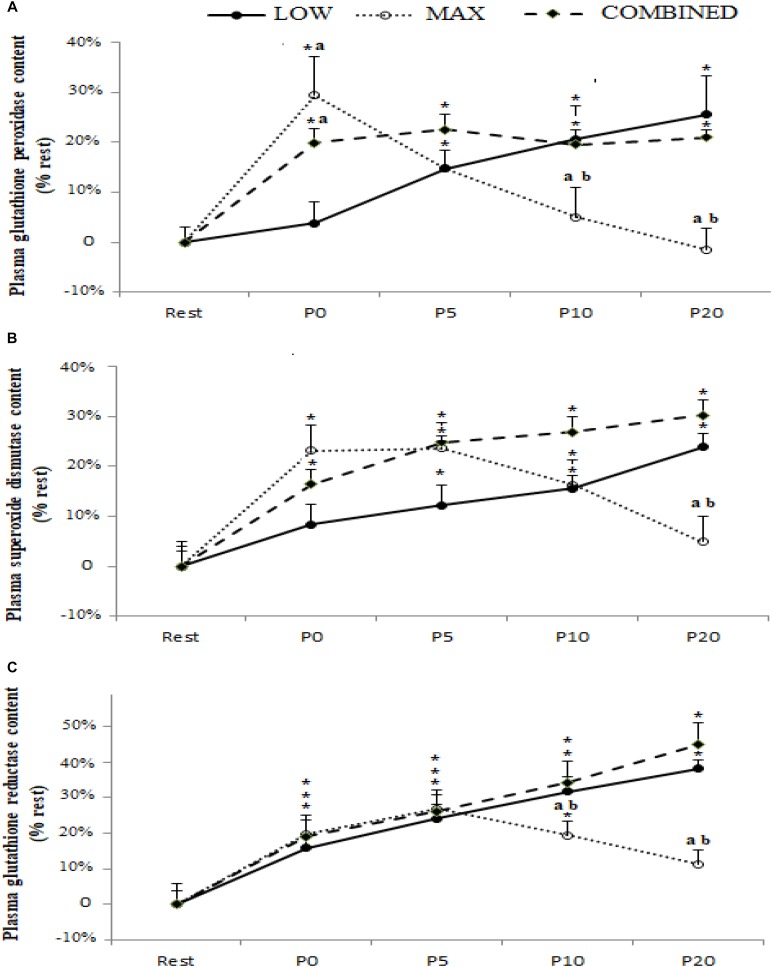
Plasma glutathione peroxidase **(A)**, superoxide dismutase **(B)** and glutathione reductase **(C)** content before (Rest), immediately after (P0), and 5 (P5), 10 (P10) and 20 (P20) minutes after maximal-intensity, low-intensity and maximal- and low-intensity exercise combined. Data are expressed as the % change from pre-exercise resting concentrations. LOW: low-intensity exercise; MAX: maximum-intensity exercise; COMBINED: combined maximum-intensity and low-intensity exercise; ^∗^: significant difference compared to pre-test values; a: significant difference compared to the LOW exercise, b: significant difference compared to the COMBINED exercise.

**FIGURE 3:**
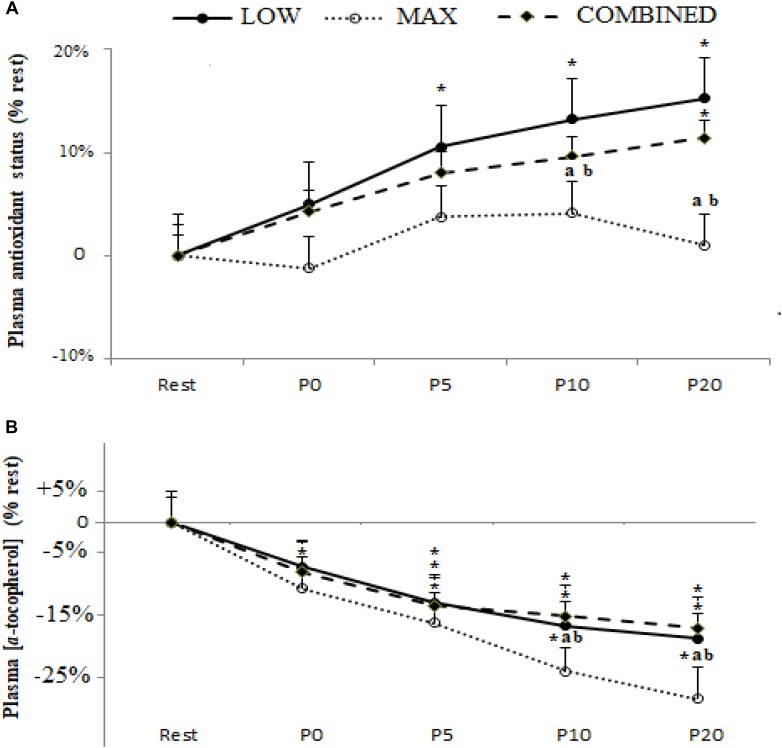
Plasma antioxidant status **(A)** and α-tocopherol concentration **(B)** before (Rest), immediately after (P0), and 5 (P5), 10 (P10) and 20 (P20) min after maximal-intensity, low-intensity and maximal- and low-intensity exercise combined. Data are expressed as the % change from pre-exercise resting concentrations. LOW: low-intensity exercise; MAX: maximum-intensity exercise; COMBINED: combined maximum-intensity and low-intensity exercise; ^∗^: significant difference compared to pre-test values; a: significant difference compared to the LOW exercise, b: significant difference compared to the COMBINED exercise.

All statistical analyses were performed using STATISTICA 10.0 Software. Normality of the data distribution was confirmed using the Shapiro-Wilk’s test. To analyze the acute effect of exercise type on oxidative stress biomarkers a two-way repeated-measures ANOVA exercise type (3 levels: MAX, LOW and COMBINED) × time (5 levels: rest, P0, P5, P10, and P20) was utilized. *Post hoc* tests were conducted using Fisher’s least significant difference (LSD). Effect sizes (ES) were calculated using partial eta-squared (ηp^2^) and magnitudes were interpreted using the thresholds: ES < 0.2 was considered small, ES around 0.5 was considered medium and ES > 0.8 was considered large (Hopkins, 2012). Statistical significance was set at *P* < 0.05 and data are presented as mean ± SE (Figure 1–3 and Supplementary Table S1).

## Results

Plasma [MDA] pre- and post-test for the MAX, LOW and COMBINED conditions is presented in Figure 1. There was a significant exercise-type × time interaction effect for plasma [MDA] [*F*_(8,72)_ = 3.51, *p* = 0.0017 and η_*p*_^2^ = 0.28]. Plasma [MDA] increased immediately (P0) after the test session with a higher rate of increase during the MAX (25.6 ± 7.8%) and LOW (23.2 ± 5.7%) conditions compared to the COMBINED (10.8 ± 3.6%) condition (*p* < 0.05). Higher MDA concentrations (*p* < 0.05) were registered at P5, P10 and P20 compared to P0 only following the LOW exercise. Moreover, [MDA] was higher at P10 and P20 in the LOW condition compared to the MAX and COMBINED conditions (*p* < 0.05; Figure 1).

Blood antioxidant enzyme content in response to the LOW, MAX and COMBINED protocols are presented in Figure 2. A significant exercise-type × time interaction was registered for GPX [*F*_(8_*_._*_72)_ = 5.79, *p* = 0.0004, ηp^2^ = 0.40], SOD [*F*_(8_*_._*_72)_ = 3.16, *p* = 0.004, ηp^2^ = 0.26] and GR [*F*_(8_*_._*_72)_ = 2.99, *p* = 0.006, ηp^2^ = 0.25] content. Regardless of the exercise type, blood GR content increased immediately after exercise (*p* < 0.05). The blood content of GPX and SOD, both increased immediately post-exercise in the MAX and COMBINED conditions compared to the resting baseline (*p* < 0.05). However, GPX and SOD content was not increased above the resting baseline in the LOW condition until 5 min post-exercise (*p* < 0.05). Compared to the MAX condition, LOW and COMBINED exercise resulted in greater GPX and GR content at P10 (*p* < 0.05) and P20 (*p* < 0.01), and a greater SOD content at P20 (*p* < 0.05). Following MAX exercise, GPX content had returned to baseline values at P10 (*p* < 0.05), whereas SOD and GR content did not return to baseline values until the P20 sampling point (*p* > 0.05). Blood GPX, SOD and GR content remained elevated above the resting baseline in the LOW and COMBINED conditions (*p* < 0.05).

Plasma TAS and [α-tocopherol] following the MAX, LOW and COMBINED exercise protocols are shown in Figure 3. There was a significant main effect for time for TAS [*F*_(4,36)_ = 3.47,*p* = 0.017,η_*p*_^2^ = 0.29] and [α-tocopherol] [*F*_(4,36)_ = 7.86, *p* = 0.0001, ηp^2^ = 0.46]. Compared to pre-exercise values, an increase in TAS was only registered following the LOW exercise protocol at P5 (*p* < 0.05). Plasma [α-tocopherol] concentration was lower at P0 (*p* < 0.05) following only MAX exercise and at P5, P10 and P20 following the three types of exercise (*p* < 0.05). Both TAS and [α-tocopherol] were lower following MAX exercise compared to LOW and COMBINED exercise at both P10 (*p* < 0.05) and P20 (*p* < 0.01). TAS levels had returned to baseline values at P20 only following MAX exercise (*P* > 0.05).

There was no significant effect of exercise type on post-exercise RPE (15.7 ± 0.9 for LOW, 15.9 ± 1.1 for MAX and 16.3 ± 0.7 for COMBINED; *p* > 0.05).

## Discussion

The present study assessed a series of oxidative stress biomarkers prior to and following MAX, LOW and COMBINED exercise. Plasma [MDA] and TAS, and blood GPX, SOD and GR increased, and plasma [α-tocopherol] declined, post-exercise in all exercise protocols, consistent with the development of exercise-induced oxidative stress. However, the principal original findings of the current study were: (1) blood GPX and SOD content increased above baseline immediately post-exercise in the MAX and COMBINED protocols, but not the LOW protocol; (2) blood GPX, SOD, GR and plasma TAS were lower 20 min post-exercise in the MAX compared to the LOW and COMBINED protocols; and (3) plasma [MDA] was highest 20 min following the LOW protocol and plasma [α-tocopherol] was lowest 20 min following the MAX protocol. These findings offer insight into exercise-type-specific oxidative stress development and might have implications for improving understanding of skeletal muscle fatigue, recovery and adaptive remodeling in response to discrete exercise protocols.

In the present study, plasma [MDA], GPX, SOD, GR and TAS increased, and [α-tocopherol] declined, post-exercise in the MAX, LOW and COMBINED exercise protocols. These observations are consistent with the development of exercise-induced oxidative stress, and in line with numerous previous studies reporting increased oxidative stress biomarkers after different types of exercise (Ammar et al., 2015a, 2016a, 2017b; Wadley et al., 2016; Parker et al., 2018).

While there is strong evidence to support the development of exercise-induced oxidative stress, it is less clear how different types of exercise influence the degree of oxidative stress. Indeed, existing studies assessing the effects of different exercise protocols on biomarkers of oxidative stress development have yielded equivocal findings (Marzatico et al., 1997; Bloomer et al., 2005; Parker et al., 2018). Baker et al. (2004) and Child et al. (1998) showed a significant increase in oxidative stress responses following low-intensity aerobic exercise, while other studies (Niess et al., 1996; Margaritis et al., 1997) have reported no pre- to post-exercise changes in [MDA]. Similarly, high-intensity anaerobic (Baker et al., 2004) and combined low- and high-intensity exercise (Thompson et al., 2003; Bloomer et al., 2006; Ascensão et al., 2008) have been reported to either increase pre- to post-exercise [MDA] or have no significant effect on pre- to post-exercise [MDA] (Svensson et al., 1999; Bloomer et al., 2006). In part, these discrepancies might be linked to inter-study differences in participant characteristics, exercise protocols and the series of biomarkers employed to assess oxidative stress. To limit the influence of these confounding variables, the present study assessed the effects of three different exercise protocols on the development of oxidative stress, inferred from the same set of oxidative stress biomarkers, in the same participants. In the present study, plasma [MDA] was increased above the pre-exercise baseline in the MAX, LOW and COMBINED exercise protocols immediately post-exercise with low post-exercise values during COMBINED protocol and no differences between the LOW and MAX protocols. These observations are consistent with some (Ascensão et al., 2008; Ammar et al., 2015a, 2016a), but not all (Svensson et al., 1999; Bloomer et al., 2006) previous studies reporting an increased [MDA] immediately post-exercise, with no difference between aerobic and anaerobic based exercise (Bloomer et al., 2005). However, in the present study plasma [MDA] was higher in the LOW protocol compared to the MAX and COMBINED protocols 10- and 20-min post-exercise.

Concerning antioxidant defense biomarkers, it was previously suggested that in response to an increased production of free radicals, concentrations of antioxidant enzymes may increase to counteract the elevated radical production and thereby minimize oxidative damage (Bloomer and Goldfarb, 2004; Ammar et al., 2015a). The present findings confirm this suggestion and showed that the blood content of the antioxidant enzymes, GPX, SOD and GR, as well as TAS, increased post-exercise in the MAX, LOW and COMBINED exercise protocols. These responses have been attributed to an increased production of ROS and a resultant increase in content of antioxidant enzymes to attenuate the development of exercise-induced oxidative stress (Bloomer, 2008; Ammar et al., 2015a, 2016a). The findings of the current study are in line with some previous studies reporting increased enzymatic antioxidant activities immediately following short-duration high-intensity exercise, such as 100 m swimming (Inal et al., 2001) and 6 × 150 m sprints (Marzatico et al., 1997), and longer duration low-intensity running (Ji, 1993) or swimming (Inal et al., 2001) exercise, and extend these observations by revealing an increase in the content of key antioxidant enzymes in the blood following the COMBINED exercise protocol. However, our observations conflict with some previous studies which reported no increase in antioxidant enzyme content following a Wingate test (Groussard et al., 2003). These inter-study discrepancies may reflect the higher training status of the elite athletes assessed in the present study. Indeed, it has been reported that increased activation of redox-sensitive transcription factors (e.g., NF-κB) in well-trained individuals can improve the production of endogenous antioxidants in response to physical exercise (Cuevas et al., 2005).

An important novel observation from the current study was the divergent response dynamics of the antioxidant biomarkers assessed across the different exercise protocols administered. Indeed, the content of SOD and GPX, but not GR and TAS, was higher immediately post-exercise in the MAX and COMBINED protocols compared to the LOW protocol. However, at the P20 time point, SOD, GPX, GR and TAS were lower in the MAX compared to the LOW and COMBINED protocols. Collectively, these plasma biomarkers pertaining to antioxidant status suggest that exercise incorporating short-duration maximal exercise can expedite the increase in some systemic antioxidant processes compared to low-intensity longer duration exercise. Conversely, 20 min following the cessation of exercise, antioxidant biomarkers were elevated during exercise incorporating 30 min of low-intensity exercise compared to shorter duration maximal intensity exercise. These observations are consistent with some evidence that post-exercise oxidative stress biomarkers are impacted by exercise intensity and duration (Ji, 1993; Parker et al., 2014), with the findings of the current study suggesting that maximal duration exercise elicits a more rapid but transient increase in antioxidant processes compared to a slower responding but longer lasting change in antioxidant processes following longer duration and lower intensity.

With regard to the interplay between the markers pertaining to antioxidant status (SOD, GPX, GR and TAS) and ROS-mediated oxidation (plasma [MDA] and [α-tocopherol]), it is possible that the greater increase in SOD and GPX content immediately post-exercise in the MAX compared to the LOW protocol could be linked to enhanced ROS-mediated oxidation in the former. Indeed, plasma [α-tocopherol] declined to a greater extent immediately following the MAX compared to the LOW protocol, which may have resulted in a compensatory up-regulation in antioxidant processes, including blood SOD and GPX content, to limit exercise-induced oxidative stress development. On the other hand, plasma [MDA] was not different immediately following the MAX and LOW protocols. Conversely, 20 min following the completion of the MAX protocol, SOD, GPX, GR and TAS were lower than the LOW protocol concomitant with a lower plasma [MDA]. Therefore, lowering in antioxidant processes 20 min post the MAX protocol could reflect a lowering in ROS-mediated oxidation and, by extension, a lesser requirement to increase antioxidant processes to maintain an optimal cellular redox balance. However, plasma [α-tocopherol] was lower 20 min following the MAX compared to the LOW protocol. Therefore, further research is required to resolve the mechanisms for the changes in, and interplay between, post-exercise prooxidant and antioxidant biomarkers and how these markers relate to exercise performance, recovery and adaptation. Moreover, since oxidative stress biomarkers were only assessed for up to 20 min post-completion of the exercise protocols administered in the current study, further research is required to assess oxidative stress biomarkers over a longer period until all biomarkers have returned to baseline to provide a more complete picture of exercise-induced oxidative stress.

### Experimental Considerations

To the best of the authors’ knowledge, this is the first study to provide information regarding the effect of different forms of exercise on oxidative stress responses in judo athletes. However, while the findings of the present study indicate exercise intensity might be a key determinant of the redox perturbation evoked by exercise, a limitation of the current study is that the three exercise tests administered were not work-matched. Accordingly, and despite reporting a similar post-exercise RPE, the design of present study did not allow us to separate the effects of exercise intensity from exercise duration when the same amount of work was completed. Additionally, the current study is based on a small sample of participants which is not enough to allow for generalization. Therefore, further research investigating a larger sample of participants is required to improve understanding of the influence of exercise intensity, duration and their interaction on exercise-induced redox perturbations.

## Conclusion

In conclusion, compared to baseline values [MDA], GPX, SOD and GR and TAS increased, and [α-tocopherol] declined, following the completion of the MAX, LOW and COMBINED exercise protocols administered in the current study. While these observations are consistent with the development of exercise-induced oxidative stress, the principal original findings from this study pertain to the characterization of exercise-type-specific oxidative stress development. Specifically, SOD and GPX content were increased immediately post-exercise, and GPX, SOD and GR and TAS were lower 20 min post-exercise, in the MAX compared to the LOW and COMBINED exercise protocols. These findings suggest that some antioxidant defense processes increase and subsequently return to baseline more rapidly after MAX exercise. However, the exercise-type-specific effect on prooxidant biomarkers is complicated by our observations that the increase in plasma [MDA] was greatest following LOW exercise and the largest decline in plasma [α-tocopherol] occurred following MAX exercise. These original findings provide insight into exercise-type-specific oxidative stress development which might have implications for exercise performance, recovery and adaptation.

## Data Availability

The datasets generated for this study are available on request to the corresponding author.

## Ethics Statement

Participants provided written informed consent to participate in the study. The study was conducted according to the declaration of Helsinki with the protocol fully approved by the Sfax University Ethics Committee before the commencement of the study.

## Author Contributions

KEA, AA, and AH conceived and performed the experiment. KEA, AA, OB, LM, SB, AH, and NB drafted and critically revised the manuscript. All authors approved the final version.

## Conflict of Interest Statement

The authors declare that the research was conducted in the absence of any commercial or financial relationships that could be construed as a potential conflict of interest.

## Supplementary Material

TABLE S1 Plasma levels of all tested parameters (mean ± SE) before (Rest), immediately after (P0), and 5 (P5), 10 (P10), and 20 (P20) min after maximal-intensity, low-intensity, and maximal- and low-intensity exercise combined.
